# Omalizumab for Urticarial Vasculitis: Case Report and Review of the Literature

**DOI:** 10.1155/2015/576893

**Published:** 2015-09-08

**Authors:** Misbah Nasheela Ghazanfar, Simon Francis Thomsen

**Affiliations:** ^1^Department of Dermatology, Bispebjerg Hospital, 2400 Copenhagen NV, Denmark; ^2^Center for Medical Research Methodology, Department of Biomedical Sciences, University of Copenhagen, 2200 Copenhagen N, Denmark

## Abstract

Urticarial vasculitis is characterised by inflamed itching or burning red patches or wheals that resemble urticaria but persist for greater than 24 hours. It is often idiopathic but is sometimes associated with collagen-vascular disease, particularly systemic lupus erythematosus. Treatment options include oral antihistamines, oral corticosteroids, dapsone, colchicine or hydroxychloroquine. We describe a male patient with urticarial vasculitis who was treated with omalizumab (anti-IgE) with convincing results and provide a review of previous reports of patients with urticarial vasculitis treated with omalizumab.

## 1. Introduction

Urticarial vasculitis is a variation of cutaneous vasculitis. Individual lesions appear as inflamed itching or burning red patches or wheals that resemble urticaria but persist for greater than 24 hours. Histopathologically, urticarial vasculitis presents as leukocytoclastic vasculitis with a perivascular mixed infiltrate of lymphocytes, neutrophils, and eosinophils, as well as fibrin deposits. Urticarial vasculitis is classified as normocomplementaemic or hypocomplementaemic based on the level of complement protein in blood. Although both subtypes are associated with typical symptoms such as angioedema, chest or abdominal pain, fever, and joint pain, the symptoms are more prominent in the hypocomplementaemic form, which is associated with systemic lupus erythematous. The majority of urticarial vasculitis cases are idiopathic [1, 2].

Recommended treatments for urticarial vasculitis are oral antihistamines and systemic immunosuppressant drugs such as oral corticosteroids, dapsone, colchicine, or hydroxychloroquine. While oral antihistamines might be useful for symptomatic relief of itch and for mild cutaneous disease without systemic involvement, most patients will need a course of oral corticosteroids to control exacerbation of cutaneous or systemic symptoms. Patients with hypocomplementaemic urticarial vasculitis with or without systemic lupus erythematous may have a more favourable response to dapsone, but the mechanism of action of dapsone is poorly understood and there are several adverse effects such as haemolysis, severe headache, and agranulocytosis. Monoclonal antibodies such as omalizumab (anti-IgE) have also been suggested for treatment of urticarial vasculitis [3]. An effective treatment for urticarial vasculitis is much needed as urticarial vasculitis impacts negatively the quality of life.

Omalizumab is a humanized anti-IgE monoclonal antibody that has recently been approved for treatment of chronic urticaria. Studies have shown that omalizumab significantly reduces the activity and symptoms of chronic urticaria. Also, omalizumab reduces the need for additional medication and improves quality of life. Clinical phases II and III studies have concluded that the ideal omalizumab dose for the treatment of chronic urticaria is 300 mg s.c. administered once every four weeks [4–6].

Herein, we describe a male patient with urticarial vasculitis who was treated with omalizumab with convincing results. Furthermore, we review previous reports of patients with urticarial vasculitis treated with omalizumab.

## 2. Case Presentation

**Figure 1:**
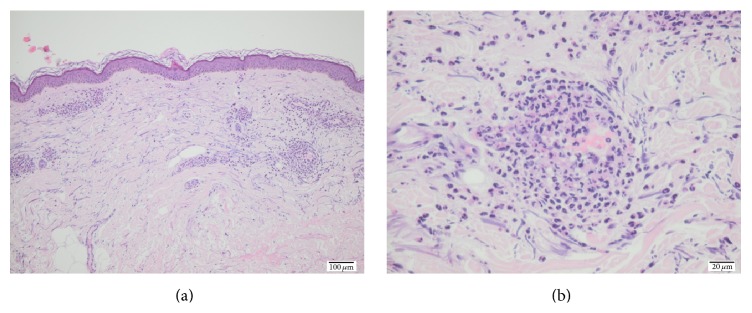
Histopathological sections of the patient showing leukocytoclastic vasculitis.

The patient was a 68-year-old man who unexpectedly developed severe, burning skin rashes clinically typical of urticarial vasculitis on his trunk, proximal upper extremities, and lower extremities during June 2014. The rashes consisted of erythematous and violaceous slightly ecchymotic infiltrated annular wheals lasting for more than 24 hours that resolved with slight postinflammatory hyperpigmentation. The patient had no angioedema and no extracutaneous manifestations such as fever, arthralgia, lymphadenopathy, uveitis, or serositis. Shortly before the onset of symptoms, he had a toe infection and received a short course of penicillin. The patient had a history of previous colon cancer for which he underwent surgery in 2008. Furthermore, he had total knee replacement surgery in 2012 due to arthritis, but he had no other previous history of rheumatic or collagen-vascular disease. He had basal cell carcinoma on the forehead in 2013. Routine blood tests including anti-cytoplasmic antibodies and anti-nuclear antibodies were normal, but he had slightly elevated secretion of protein in the urine. A skin biopsy revealed leukocytoclastic vasculitis with perivascular infiltrates primarily of neutrophils (Figure 1).

The patient was treated with oral prednisolone at a dose of 37.5 mg once daily for one week and an improvement was observed. The patient was then advised to reduce the dosage of prednisolone to 25 mg daily for three days and then to 12.5 mg daily for one week and then slowly taper prednisolone altogether. After three months, the patient experienced relapse of skin rashes and was then treated with dapsone 50 mg twice daily for one month but without significant improvement. In December 2014, the patient was switched to omalizumab 300 mg s.c. once every four weeks, and already after one month a complete remission of the urticarial vasculitis and symptoms was observed. By July 2015, the patient is still being treated with omalizumab 300 mg s.c. every four weeks with sustained remission and no apparent adverse effects. The patient was not treated with oral antihistamines throughout the course of the disease.

## 3. Discussion

Clinical phase studies have shown that omalizumab is safe and reduces disease activity in patients with chronic urticaria [5, 6]. While there are no prospective clinical studies of omalizumab for urticarial vasculitis, a few case reports have shown that omalizumab might also be beneficial for this indication (Table 1). A case report by Del Pozo et al. described a female patient with systemic lupus erythematous and urticarial vasculitis. She was treated with oral antihistamines, oral corticosteroids, and azathioprine without improvement. She was then administrated omalizumab based on weight and IgE levels, which showed great improvement as her lesions disappeared [3]. Another case by Varricchi et al. described a female patient with asthma, Churg-Strauss syndrome, and urticarial vasculitis. She also had no symptomatic improvement while being treated with oral antihistamines, oral corticosteroids, or other immunosuppressants such as azathioprine and cyclosporine. She was then treated with omalizumab 300 mg s.c. every two weeks as an add-on to antihistamines, oral corticosteroids, and immunosuppressants. After 6 months of treatment, the patient reported a significant improvement of her condition [7]. A Spanish case report involving three female patients with chronic spontaneous urticaria with autoimmune and pressure components and vasculitis also reported successful treatment with omalizumab [8]. Finally, an open-label study by Sussman et al. of patients with chronic urticaria involving also one patient with urticarial vasculitis noted that omalizumab was a sufficient treatment for the patients included in the study [9]. None of the patients included in these case studies experienced serious adverse effects during treatment.

Urticarial vasculitis with eruptive erythematous wheals resembles chronic urticaria, but the individual lesions usually last longer than in chronic urticaria. Symptoms are more frequently burning rather than itching and resolve with hyperpigmentation [1]. Several clinical studies have shown that omalizumab has great effect on chronic urticaria [5, 6] and while omalizumab is not the choice of treatment for urticarial vasculitis, it seems to have a beneficial effect on patients with urticarial vasculitis as reported herein and in the earlier published case reports. However, the mechanisms of action of omalizumab for urticarial vasculitis remain, in part, unresolved. Particularly, it is not known whether omalizumab is efficacious against both normocomplementaemic and hypocomplementaemic urticarial vasculitides. We did not measure levels of complement in blood in our patient.

## 4. Conclusion

Our report of successful treatment of urticarial vasculitis with omalizumab is important, as many patients with urticarial vasculitis cannot be treated successfully or experience significant side effects of the standard treatment. However, clinical trials with a greater number of patients with urticarial vasculitis that compare standard treatment with omalizumab are warranted.

## Figures and Tables

**Table 1:** Patients with urticarial vasculitis treated with omalizumab.

Author	Year	Case	Dose of omalizumab	Effect of omalizumab
Del Pozo et al. [3]	2012	Female, aged 51, with SLE and urticarial vasculitis. No improvement with oral corticosteroids, antihistamines, and azathioprine	Unknown: based on weight and IgE	Significant improvement Lesions disappeared. No hives or pain

Varricchi et al. [7]	2012	Female, aged 44, with asthma, Churg-Strauss syndrome, and urticarial vasculitis. No improvement with oral corticosteroids, antihistamines, azathioprine, and cyclosporine	300 mg s.c. every two weeks	Significant improvement of symptoms

Díez et al. [8]	2013	Three females with chronic spontaneous urticaria with autoimmune and pressure components plus vasculitis. No improvement with antihistamines, leukotriene receptor antagonists, and cyclosporine	150 mg s.c. every four weeks (two patients) and 300 mg s.c. every four weeks (one patient)	Remission of symptoms in all three patients

Sussman et al. [9]	2014	One patient, unknown gender and age, with urticarial vasculitis	150 mg s.c. every four weeks	Remission of symptoms. Details not given
